# Prior Out-of-Home Placement and Length of Stay Among Youths Receiving Mental Health Services in the ED

**DOI:** 10.1001/jamanetworkopen.2025.55339

**Published:** 2026-01-23

**Authors:** Casey K. Kelly, Maria Saliba, Jin Hong Park, Helder K. Yoshii, Idil Tarikogullari, Abby Tarasewicz, Alexandra Kaase, Caleb Porter, Stacey M. Boehm, Sydney N. Loy, Allison LeMahieu, Magdalena Romanowicz, Monica J. Taylor-Desir

**Affiliations:** 1 Department of Psychiatry & Psychology, Mayo Clinic, Rochester, Minnesota; 2 Department of Pediatrics, Mayo Clinic, Rochester, Minnesota; 3 Department of Quantitative Health Sciences, Mayo Clinic, Rochester, Minnesota

## Abstract

**Importance:**

Youth mental health crises have been increasing over the last decade, and there is an urgent need for clinicians to be more knowledgeable about patients with high emergency department (ED) utilization. Several disparities in ED utilization and outcomes have already been identified; however, little data exist on disparities affecting youths with histories of out-of-home placement (OOHP).

**Objective:**

To explore whether history of OOHP is associated with increased length of stay among child and adolescent patients who present to the emergency department with psychiatric symptoms.

**Design, Setting, and Participants:**

This retrospective, electronic health record (EHR)–based, cross-sectional study included patients aged 17 years or younger with a child and adolescent psychiatric consultation placed in the Mayo Clinic Rochester ED between January 1, 2021, and June 30, 2024. The Mayo Clinic Rochester is a tertiary referral center that serves as a regional hub for both primary and specialized psychiatric care.

**Main Outcomes and Measures:**

The primary outcome was the length of stay in the ED. Secondary outcomes were use of physical and pharmacological restraint. Associations between OOHP and length of stay were examined via linear mixed-effects regression models with length of stay log transformed.

**Results:**

Of the 1572 care encounters (median [IQR] age, 14,9 [13.3-16.3] years) among 1119 unique patients, there were 1244 with no history of OOHP and 328 with history of OOHP. Among the OOHP group, 158 (48%) were male and 170 (52%) female; 11 (4%) American Indian or Alaska Native, 7 (2%) Asian, 49 (16%) Black, 43 (14%) Hispanic, and 222 (71%) White. Among the 1244 encounters without OOHP, 820 (66%) were among female patients and 423 (34%) male; 21 (2%) American Indian or Alaska Native, 47 (4%) Asian, 121 (10%) Black, 125 (10%) Hispanic, and 971 (80%) White. Children and adolescents with history of OOHP were observed to spend 24% (95% CI, 12%-36%) more time in the ED even when adjusting for age at admission, sex, insurance, number of prior diagnoses, presenting concerns, and reasons for prolonged boarding (*P* = .004). Children and adolescents with history of OOHP had 2.05 (95% CI, 1.69-2.48) higher odds of being physically restrained (*P* < .001) and 2.15 (95% CI, 1.79-2.58) higher odds of receiving pharmacologic restraints (*P* < .001) while in the ED.

**Conclusions and Relevance:**

In this cross-sectional study of 1572 care encounters among 1119 patients, history of OOHP was associated with longer lengths of stay in the emergency department for children and adolescents who presented for mental health concerns. The findings highlight the need for further research on ways to mitigate the risk of extended emergency department stays for children with OOHP.

## Key Points

**Question:**

Is history of out-of-home placement (OOHP) associated with length of stay or use of physical or pharmacologic restraints among youths receiving mental health care in the emergency department?

**Findings:**

In this cross-sectional study of 1572 care encounters among 1119 patients, those with history of OOHP placement (328 encounters) spent approximately 24% more time in the ED vs those without history of OOHP. These patients also had higher odds of receiving physical or pharmacologic restraints for agitation.

**Meaning:**

The findings of this study highlight the need for specialized, culturally sensitive, and trauma-informed services in psychiatric emergency settings for youths with a history of OOHP.

## Teaser

This cross-sectional study evaluates whether youth history of out-of-home placement (OOHP), ie, foster care, is associated with length of stay among children and adolescents who receive mental health care at an emergency department (ED).

## Introduction

Mental health disorders among youths have been increasing over the last decade, with emergency department (ED) visits for self-harm assessments increasing by 329% and all pediatric mental health assessments in the ED increasing by 60%.^1,2^ In 2022, the culmination of these circumstances led to the US Surgeon General declaring a mental health crisis for children and adolescents. The excessive demand on the mental health care system has manifested most significantly in EDs through “boarding,”^3^ where patients experience prolonged stays in the ED while awaiting transfer to a suitable treatment facility. The Joint Commission defines boarding as “the practice of holding patients in the ED or another temporary location after the decision to admit or transfer has been made”^4^ and recommends boarding duration of less than 4 hours. Boarding of ED patients with mental health and behavioral issues decreases the already limited number of pediatric beds for medical and surgical patients.

Children in out-of-home placement (OOHP), also known as foster children, are known to have high ED utilization^5,6^ and account for nearly half a million children in the United States annually.^7^ OOHP significantly increases the risk of developing mental and physical health concerns as well as premature death. In psychiatrically hospitalized children, multiple OOHPs was the most consistent correlate of externalizing problems (eg, rule-breaking behavior, aggression, and impulsivity) and internalizing problems (eg, worry, anhedonia, self-criticism, and somatic concerns) as well as psychosocial impairment.^8^ OOHPs disrupt stability, safety, and security in an already vulnerable developing population. Placement instability is associated with decreased rates of permanency and increased rates of behavioral problems and disruption in emotional regulation in these children and adolescents.^9^

The culmination of these challenges is that many children and adolescents with a history of OOHPs end up in the ED, becoming trapped in a cycle of lengthy ED stays and/or hospitalizations, housing and guardianship changes, and escalating responses to stressors disrupting the continuity of care that is essential to their well-being. Despite this, there have been minimal studies on OOHP in pediatric psychiatric emergency settings.

The objective of the study was to quantify whether a history of OOHP was associated with increased length of stay in the ED when presenting with mental health concerns. We anticipated that patients with a history of OOHP would be observed to spend increased time in the ED compared with those without OOHP history. We also conducted exploratory analyses to examine whether OOHP history was associated with other adverse outcomes during ED visits (eg, use of physical restraints and pharmacologic restraints).

## Methods

### Study Population

A retrospective cross-sectional analysis was performed by reviewing the electronic health record (EHR) of children and adolescents aged 0 to 17 years who presented to Saint Mary’s ED at Mayo Clinic in Rochester, Minnesota, and for whom a psychiatric consultation was requested by the primary emergency medicine team. Data were collected from January 1, 2021, to June 30, 2024.

Participants were included in the study if they met the following criteria: (1) aged 17 years or younger and (2) had parent or guardian consent for research participation. Exclusion criteria included (1) those without parent or guardian consent and (2) children who were currently in OOHP, without a history of OOHP. A total of 1572 encounters were identified from 1119 patients for inclusion based on the retrospective review of medical records (Figure).

**Figure.**
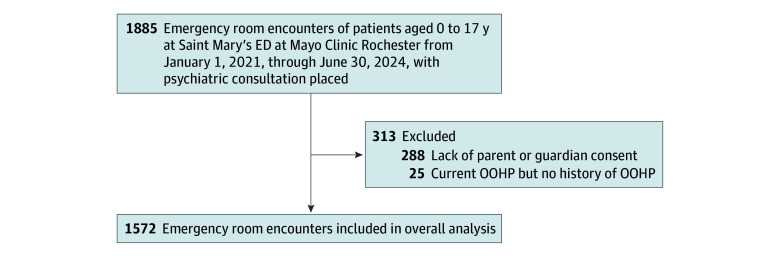
Patient Selection ED indicates emergency department; OOHP, out-of-home placement.

This retrospective study involved review of electronic medical records for patients who were minors at the time of clinical care. In accordance with Mayo Clinic policy, only records of minors whose parent or legal guardian had previously granted Mayo Clinic Research Authorization were included. Medical records of minors whose parent or guardian declined authorization were excluded from all analyses. All protected health information was handled in compliance with Mayo Clinic institutional review board requirements, including strict confidentiality protections and secure data management procedures. The study adhered to the Strengthening the Reporting of Observational Studies in Epidemiology (STROBE) reporting guideline.

### Study Measures

Demographic data, including age, natal sex, race, ethnicity, and insurance, which are inputted into the EHR via patient self-report, were collected. The primary outcome of interest was the length of stay in the ED, which was defined by time of ED admission or arrival to time at ED discharge. ED discharge locations were also collected, including medical hospital, inpatient psychiatric hospital, residential treatment center, juvenile detention center, foster care, home with family, and other. Secondary outcomes included physical restraint and pharmacologic restraint use. These were identified by corresponding orders in the ED encounter. Medications were considered to be pharmacologic restraints if they were (1) administered intravenously or intramuscularly and (2) previously cited in literature as being used for restraint in pediatric populations.^10^ OOHP was collected by standardized medical record abstraction using REDCap, where OOHP represents a child that has been in the physical custody of someone other than the child’s parent. The determination of OOHP was made by the study team based on information documented in the medical record. In most cases, OOHP status was clearly indicated in the social history section of the medical record. If it was not explicitly stated, a targeted keyword search of the entire medical record was conducted using predefined OOHP criteria (eg, foster care, adoption placement, kinship care, group home, residential facility, juvenile detention) to ensure consistent identification across all patients. Any ambiguous cases were reviewed by 2 team members, and consensus was reached through discussion. If a patient was adopted from birth, this was not considered OOHP. Those who were documented as being in OOHP for the first time at the index ED admission, with no prior history of OOHP noted in any earlier encounters, were included as a separate subgroup. Because this subgroup was small (25 encounters) (eTable 1 in Supplement 1) and because their OOHP status represented a new onset rather than an established history, we did not include them in the OOHP analysis groups. Our primary comparison focused on patients with a documented history of OOHP prior to the index visit, allowing us to examine the association between prior OOHP exposure and ED outcomes.

Additional clinical data variables, including presenting concern, primary psychiatric diagnoses, number of previous psychiatric hospitalizations, active psychiatric medications, substance use, discharge destination, legal guardian at the time of ED encounter, legal history, history of sexually intrusive behaviors, and history of trauma, were manually extracted from the EHR. Of note, some patients had arrived to the ED with more than 1 presenting concern, and this was collected as such. History of trauma was defined as any self-report or any mandated reporting of maltreatment that was documented in the EHR prior to the ED encounter. This was further delineated into subtypes including family conflicts, physical abuse, neglect, medical trauma, sexual trauma, homelessness, emotional abuse, verbal abuse, war trauma, natural disaster, and other. In ED encounters that were greater than 4 hours from the time of psychiatric consult, an attempt to characterize the reason for boarding was made. This was extracted from case management notes, which are used in the process of making referrals to outside hospitals. In many cases, more than one reason was identified; these data are displayed in eFigure in Supplement 1. eTable 2 in Supplement 1 shows the number of admissions vs discharges in those with and without OOHP.

### Statistical Analysis

Patient characteristics were summarized using median (IQR) for continuous variables and frequency (percentage) for categorical variables. These characteristics are presented overall and separately based on the history of OOHP. For comparison by history of OOHP, Kruskal-Wallis rank sum tests were used for continuous characteristics and Pearson χ^2^ tests for categorical characteristics.

To satisfy distributional assumptions, length of ED stay was analyzed using log transformation. Unadjusted and adjusted linear mixed-effects regression models were used to assess the association between history of OOHP and length of ED stay, with a random intercept to account for patients with repeated ED stays. Covariates in the adjusted models included age at admission, sex, insurance, primary psychiatric diagnoses, number of psychotropic medications, number of psychiatric hospitalizations, history of trauma (yes or no), presenting concerns, and reasons for prolonged boarding. Subsetting to patients with history of OOHP, unadjusted and adjusted linear mixed-effects regression models analyzed the association between race and ethnicity and length of ED stay. For all analyses involving race, race categories were collapsed to Black or African American, White, and additional groups (American Indian or Alaska Native, Asian, Middle Eastern or North African, multiracial, and other). Exploratory analyses assessed the association between use of physical restraints and pharmacologic restraints with length of ED stay using unadjusted and adjusted linear mixed-effects regression models. Additional exploratory analyses assessed the association between race and ethnicity and physical or pharmacologic restraint use, regardless of OOHP history and subset to those only with a history of OOHP, using unadjusted and adjusted logistic regression models.

Missing data were limited to 5 variables (sex, race, ethnicity, health insurance, and history of trauma); all missing for less than 5% of patients. Therefore, complete case methods were applied to the missing data. A stepdown Bonferroni method (Holm)^11^ was used to account for multiple comparisons and control the family-wise error rate, with adjusted 2-tailed *P* values less than .05 considered statistically significant. For exploratory analyses, 2-tailed *P* values of .05 or less were considered to be statistically significant. Data management and statistical analysis were performed in SAS version 9.4 (SAS Institute).

## Results

We analyzed 1572 care encounters (median [IQR] age, 14,9 [13.3-16.3] years) among 1119 patients, of whom 1244 had no history of OOHP and 328 had at least 1 incident of OOHP in their lifetime. Among the history of OOHP group, a total of 158 (48%) were male, 170 (52%) were female, 11 (4%) were American Indian or Alaska Native, 7 (2%) were Asian, 49 (16%) were Black, 43 (14%) were Hispanic, and 222 (71%) were White. Among the 1244 without OOHP, 820 (66%) were female, 423 (34%) were male; 21 (2%) American Indian or Alaska Native, 47 (4%) Asian, 121 (10%) Black, 125 (10%) Hispanic, and 971 (80%) White (Table 1). Race and sex were found to be significantly different, with Black and American Indian or Alaska Native race as well as male sex being overrepresented in the history of OOHP group. Insurance status was also significantly different, with Medicaid (108 [64%]) being the most common insurance in history of OOHP group (Table 1). In both groups, the most common reasons for prolonged boarding included: bed availability, acuity or aggression, and awaiting safe disposition (eFigure in Supplement 1).

**Table 1.** Characteristics of Patients From 1572 Encounters

Characteristic	Overall, No./total. No. (%) (N = 1572)	Encounters, No. (%)		*P* value
		History of OOHP (n = 328)^a^	No history of OOHP (n = 1244)	
Age at ED admit, median (IQR), y	14.9 (13.3-16.3)	14.9 (13.3-16.3)	14.9 (13.3-16.3)	.82
Sex assigned at birth				
Female	990/1571 (63)	170 (52)	820 (66)	<.001
Male	581/1571 (37)	158 (48)	423 (34)	
Race				
American Indian or Alaska Native	32/1526 (2)	11 (4)	21 (2)	.003
Asian	54/1526 (4)	7 (2)	47 (4)	
Black or African American	170/1526 (11)	49 (16)	121 (10)	
Middle Eastern or North African	4/1526 (<1)	1 (<1)	3 (<1)	
Multiracial	7/1526 (1)	1 (<1)	6 (1)	
White	1193/1526 (78)	222 (71)	971 (80)	
Other^b^	66/1526 (4)	20 (6)	46 (4)	
Ethnicity				
Hispanic or Latino	168/1526 (11)	43 (14)	125 (10)	.07
Not Hispanic or Latino	1357/1526 (89)	266 (86)	1091 (90)	
Health insurance				
Commercial	896/1555 (58)	115 (35)	781 (64)	<.001
Medicaid	650/1555 (42)	208 (64)	442 (36)	
Uninsured	9/1555 (1)	3 (1)	6 (1)	
Pharmacologic restraints during encounter				
Any	60/1572 (4)	29 (9)	31 (3)	NA
Intramuscular	57/60 (95)	29 (100)	28 (90)	<.001
Intravenous	3/60 (5)	0	3 (10)	
Physical restraint during encounter				
Any	56/1572 (4)	21 (6)	35 (3)	NA
Physical hold	2/56 (4)	0	2 (6)	.002
Restraint chair	38/56 (68)	19 (91)	19 (54)	
Security cuff	3/56 (5)	0	3 (9)	
Soft restraints	13/56 (23)	2 (10)	11 (31)	
Presenting concern				
Suicidal ideation or self-injurious behavior	1093/1572 (70)	186 (57)	907 (73)	<.001
Suicide attempt	200/1572 (13)	27 (8)	173 (14)	.006
Violence toward others	140/1572 (9)	67 (20)	73 (6)	<.001
Elopement	44/1572 (3)	23 (7)	21 (2)	<.001
Substance use	26/1572 (2)	15 (5)	11 (1)	<.001
Hallucinations or disorganized	44/1572 (3)	13 (4)	31 (3)	.15
Other	241/1572 (15)	67 (20)	174 (14)	.004
Primary psychiatric diagnosis				
Any	1412/1572 (90)	311 (95)	1101 (89)	.001
Bipolar disorder	16/1412 (1)	9 (3)	7 (1)	.001
Psychotic disorder	16/1412 (1)	6 (2)	10 (1)	.10
Autism	112/1412 (7)	29 (9)	83 (7)	.18
Intellectual disability	20/1412 (1)	13 (4)	7 (1)	<.001
Anxiety disorder	764/1412 (49)	131 (40)	633 (51)	<.001
Depressive disorder	954/1412 (61)	166 (51)	788 (63)	<.001
ADHD	565/1412 (36)	180 (55)	385 (31)	<.001
Other	737/1412 (47)	242 (74)	495 (40)	<.001
Previous psychiatric hospitalizations, median (IQR)	0 (0-1)	1 (0-3)	0 (0-1)	<.001
Active psychiatric medications				
Stimulants	306/1572 (20)	110 (34)	196 (16)	<.001
Nonstimulants	269/1572 (17)	120 (37)	149 (12)	<.001
Typical antidepressants	844/1572 (54)	183 (56)	661 (53)	.39
Other antidepressants	119/1572 (8)	44 (13)	75 (6)	<.001
Neuroleptics	298/1572 (19)	129 (39)	169 (14)	<.001
Mood stabilizers	73/1572 (5)	49 (15)	24 (2)	<.001
Benzodiazepines	26/1572 (2)	13 (4)	13 (1)	<.001
Nonbenzodiazepines	130/1572 (8)	49 (15)	81 (7)	<.001
Substance use				
Cannabis or marijuana	474/1572 (30)	125 (38)	349 (28)	<.001
Nicotine or tobacco	335/1572 (21)	95 (29)	240 (19)	<.001
Alcohol	278/1572 (18)	74 (23)	204 (16)	.009
Cocaine, amphetamine, or stimulant	30/1572 (2)	15 (5)	15 (1)	<.001
Anxiolytic, hypnotics, or sedatives	24/1572 (2)	9 (3)	15 (1)	.04
Opioid	25/1572 (2)	13 (4)	12 (1)	<.001
Other	34/1572 (2)	15 (5)	19 (2)	.001
History of trauma, No. (%)				
Any	784/1498 (52)	259 (83)	525 (44)	<.001
Family conflicts	147/1498 (10)	70 (22)	77 (7)	<.001
Physical abuse	324/1498 (22)	144 (46)	180 (15)	<.001
Neglect	158/1498 (11)	115 (37)	43 (4)	<.001
Medical trauma	17/1498 (1)	3 (1)	14 (1)	.73
Sexual trauma	348/1498 (23)	106 (34)	242 (21)	<.001
Homelessness	9/1498 (1)	6 (2)	3 (<1)	.001
Emotional abuse	183/1498 (12)	76 (24)	107 (9)	<.001
Verbal abuse	92/1498 (6)	35 (11)	56 (5)	<.001
War trauma	0/1498	0	0	NA
Natural disaster	1/1498 (<1)	0	1 (<1)	.61
Other	100/1498 (7)	28 (9)	72 (6)	.07
Legal history	132/1572 (8)	79 (24)	53 (4)	<.001
Sexually intrusive behavior	59/1572 (4)	37 (11)	22 (2)	<.001
ED discharge location				
Medical hospital	27/1572 (2)	11 (3)	16 (1)	<.001
Inpatient psychiatric hospital	1004/1572 (64)	140 (43)	864 (70)	
Residential treatment center	15/1572 (1)	13 (4)	2 <10)	
Juvenile detention center	4/1572 (<1)	3 (1)	1 (<1)	
Foster care	5/1572 (<1)	5 (2)	0	
Home with family	414/1572 (26)	101 (31)	313 (25)	
Other	103/1572 (7)	55 (17)	48 (4)	
Legal guardian				
Mother	1258/1572 (80)	150 (46)	1108 (89)	<.001
Father	678/1572 (43)	71 (22)	607 (49)	<.001
Stepparent	32/1572 (2)	5 (2)	27 (2)	.46
Other family member	77/1572 (5)	51 (16)	26 (2)	<.001
Adoptive parent	94/1572 (6)	71 (22)	23 (2)	<.001
Case manager or county	39/1572 (3)	37 (11)	2 (<1)	<.001
Other	12/1572 (1)	7 (2)	5 (<1)	.001

Abbreviations: ADHD, attention-deficit/hyperactivity disorder; ED, emergency department; NA, not applicable; OOHP, out-of-home placement.

^a^ Excludes patients with current OOHP with no prior OOHP.

^b^ Other race is an electronic health record category that patients self-report.

Median (IQR) time spent in the ED was 15.9 (5.1-29.6) hours in the history of OOHP group compared with 4.8 (3.6-10.7) hours in the group with no history of OOHP (eTable 3 in Supplement 1). History of OOHP was associated with increased time (hours) spent in the ED, specifically 24% (95% CI, 12%-36%) longer (adjusted) (*P* = .004) (Table 2). Subsetting to only patients with history of OOHP, we found no association between race or ethnicity and duration of time in ED (Black race, adjusted estimate, 0.95; 95% CI, 0.67-1.58, *P* > .99; additional racial groups: adjusted estimate, 1.09; 95% CI, 0.74-1.58; *P* > .99; Hispanic or Latino ethnicity, adjusted estimate, 1.14; 95% CI, 0.78-1.65; *P* > .99). History of trauma was associated with total time spent in the ED (adjusted estimate, 1.09; 95% CI, 1.02-1.17; *P* = .002).

**Table 2.** Multiplicative Change in Time in the Emergency Department Based on History of OOHP and Race and Ethnicity

Characteristic	Unadjusted estimate (95% CI)^a^	*P* value	Adjusted estimate (95% CI)^a^^,^^b^	*P* value
**Among full cohort**				
OOHP (1572 encounters)	2.22 (1.96-2.51)	.004	1.24 (1.12-1.36)	.004
**Among encounters with OOHP**				
Race (reference group, White)				
Black or African American (49 encounters)	1.00 (0.64-1.55)	>.99	0.95 (0.67-1.35)	>.99
Additional groups (40 encounters)^c^	0.98 (0.61-1.57)	>.99	1.09 (0.74-1.58)	>.99
Ethnicity (reference group, not Hispanic or Latino)				
Hispanic or Latino (43 encounters)	1.61 (1.02-2.54)	.93	1.14 (0.78-1.65)	>.99

Abbreviation: OOHP, out-of-home placement.

^a^ Estimates are the multiplicative change in the time in emergency department. Data were log-transformed for normality.

^b^ Adjusted for age at admission, sex, insurance, number of prior diagnoses, number of psychotropic medications, number of psychiatric hospitalizations, history of trauma, presenting concerns, and reasons for prolonged boarding.

^c^ Additional groups includes American Indian or Alaska Native, Asian, Middle Eastern or North African, multiracial, and other race subcategories.

The use of physical restraints and pharmacologic restraints was also examined (Table 3 and Table 4). Patients with a history of OOHP had 2.05 (95% CI, 1.69-2.48) times greater odds of physical restraints being used (*P* < .001) and 2.15 (95% CI, 1.79-2.58) times greater odds of receiving pharmacologic restraints (*P* < .001) (Table 3). Black patients, regardless of OOHP history, had 4.33 (95% CI, 1.96-9.56) times greater odds of being physically restrained (*P* < .001) and 2.83 (95% CI, 1.34-5.95) times greater odds of being pharmacologically restrained (*P* = .01) (Table 4). Among those with a history of OOHP, Black patients had 4.02 (95% CI, 1.25-12.95) times greater odds of being pharmacologically restrained in the ED (*P* = .02). The association between Black race and physical restraints did not remain significant when looking only at the history of the OOHP group (eTable 4 in Supplement 1). Additional racial groups and Hispanic or Latino ethnicity were not significantly associated with physical or pharmacologic restraints in the overall group or in the history of OOHP group.

**Table 3.** Association of Physical Restraints and Pharmacologic Restraints in Encounters Among Patients With History of Out-of-Home Placement

Characteristic	Unadjusted estimate (95% CI)^a^	*P* value	Adjusted estimate (95% CI)^a^^,^^b^	*P* value
Physical restraints	4.76 (3.68-6.16)	<.001	2.05 (1.69-2.48)	<.001
Pharmacologic restraints	4.65 (3.63-5.96)	<.001	2.15 (1.79-2.58)	<.001

^a^ Estimates are the multiplicative change in the odds of receiving restraints. Data were log-transformed for normality.

^b^ Adjusted for age at admission, sex, insurance, number of prior diagnoses, number of psychotropic medications, number of psychiatric hospitalizations, history of trauma, presenting concerns, and reasons for prolonged boarding.

**Table 4.** Association of Physical Restraints and Pharmacologic Restraints and Race and Ethnicity in Youths in the Emergency Department

Race and ethnicity	Unadjusted OR (95% CI)	*P* value	Adjusted OR (95% CI)^a^	*P* value
**Physical restraints**				
Race (reference group, White)				
Black or African American	4.80 (2.56-9.01)	<.001	4.33 (1.96-9.56)	<.001
Additional groups^b^	2.52 (1.17-5.47)	.02	2.24 (0.85-5.93)	.10
Ethnicity (reference group, not Hispanic or Latino)				
Hispanic or Latino	1.65 (0.79-3.44)	.18	0.68 (0.24-1.93)	.46
**Pharmacologic restraints**				
Race (reference group, White)				
Black or African American	3.91 (2.13-7.18)	<.001	2.83 (1.34-5.95)	.01
Additional groups^b^	1.58 (0.69-3.63)	.28	1.10 (0.40-3.01)	.86
Ethnicity (reference group, not Hispanic or Latino)				
Hispanic or Latino	1.95 (0.99-3.84)	.05	1.24 (0.50-3.08)	.64

^a^ Adjusted for age at admission, sex, insurance, number of prior diagnoses, number of psychotropic medications, number of psychiatric hospitalizations, history of trauma, presenting concern(s), and reason(s) for prolonged boarding.

^b^ Additional groups includes American Indian or Alaska Native, Asian, Middle Eastern or North African, multiracial and other race subcategories.

## Discussion

Our study suggests that a history of OOHP in youth is associated with increased length of stay in the ED among patients presenting with mental health concerns. In addition, this history is associated with twice the odds of requiring physical restraints and intramuscular or intravenous pharmacologic restraints during their ED visits. Reflecting on the potential underlying reasons as to why OOHP was associated with increased length of ED stays highlights several other possible disparities. In our study, children with a history of OOHP were more likely to have had previous psychiatric hospitalizations and to be prescribed psychotropic medications, including stimulants, neuroleptics, mood stabilizers, and benzodiazepines. This might be due to the fact that they were less likely to be diagnosed with anxiety and depression and more likely to be diagnosed with externalizing disorders, including attention-deficit/hyperactivity disorder. They also were more likely to use substances during childhood or adolescence. Socially, children with a history of OOHP in the psychiatric ED were more likely to be enrolled in Medicaid, have legal history, and have experienced trauma, with the most common being physical abuse, followed by neglect and sexual trauma. This is in sync with previous research that shows that children with OOHP most commonly experience trauma in the form of physical abuse and that those children in general are at increased risk of multiple adverse childhood experiences.^12^

The association of OOHP with length of stay is likely multifactorial. In addition to the complexity of their psychiatric comorbidities and challenging psychosocial circumstances, systemic factors, such as limited bed availability, also contribute. In this study, the most common reasons for extended boarding were lack of available beds, delays in securing a safe disposition, and the need for medical clearance. Notably, we observed that children with a history of OOHP were significantly more likely to be discharged home and less likely to be transferred to an inpatient psychiatry unit. One hypothesis might be that extended stays in the ED may provide adequate time for a child’s acute psychiatric crisis to resolve without the need for inpatient hospitalization. Additionally, disparities in the ED referral process may play a role, potentially influenced by systemic biases or resource limitations. Children with OOHP are more likely to experience legal difficulties, school exclusion or expulsion, and engage in aggressive behaviors—all of which increase the likelihood of ED presentations and to make placements on inpatient psychiatry units more challenging.^13^ This seems to create a vicious cycle where children with OOHP are more likely to present to the ED, stay there longer while receiving minimal treatment, and ultimately be more likely to be discharged home to limited resources and often unstable psychosocial situations.

Fortunately, there have been a small number of studies examining ways to mitigate these risks. Ibeziako et al^14^ explored interventions that aimed to reduce the length of boarding, including expanding inpatient psychiatric beds, hiring additional staff to enhance crisis stabilization services, and initiating treatments for patients while boarding. While these interventions were not exclusively examined for children with history of OOHP, approximately 10% of their sample were living in a group home, a long-term residential facility, and/or in state custody. The interventions were found to decrease length of boarding by 53% for patients discharged to high and intermediate levels of care 1 year after full implementation.^14^ It is important to recognize that these types of interventions likely require a large up-front investment from hospitals; however, reduction of boarding allows for more efficient use of hospital beds and resources, enabling the hospital to treat more patients. Further research is needed to better understand strategies that may reduce length of stay for children with OOHP.

Our study also replicates findings on racial disparities in psychiatric emergency care. A recent small-scale study at a community hospital^15^ found higher rates of boarding among Black child and adolescent patients, as well as increased restraint use. Another recent study^16^ observed increased rates of pharmacologic restraint in Black patients in nonpsychiatric children’s hospitals. With this growing evidence, Mroczkowski et al outlined “actionable steps to address structural racism along the ED care continuum.”^17^ They highlight that the need for change extends beyond the ED and begins in community organizations, including school professionals, outpatient health care practitioners, and social agencies, that often facilitate the initial referral to the ED. Within the ED, they recommend several action items, including standardizing trauma-informed approaches to youth agitation, establishing behavioral response teams, integrating health equity into clinical education and care, and reflecting on racial biases both on individual and institutional levels. Our data emphasize the need to extend these thoughtful guidelines to youth historically impacted by OOHP.

### Limitations

Although we have attempted to minimize confounders by the study design outlined above, some inherent limitations of the study remain, largely due to its retrospective nature. As such, the study could not assess causality, as the exposure groups would not be able to be randomized. The retrospective nature of the design also introduces challenges regarding missing and erroneous data as well as biases of abstractors. We attempted to minimize these limitations by implementing a standardized data dictionary and by excluding any missing or ambiguous data from our analysis. It is important to note that our data were entirely derived from the EHR and OOHP may not always be documented in the medical record; therefore, the fidelity of our data is only as accurate and complete as these records. Alternative collection strategies, outside of medical record review, were explored to identify those with history of OOHP; however, access to Child Welfare data is highly protected, and we were unable to obtain identifiable data from Olmsted County to match to our EHR records. We acknowledge that multiple comparisons may increase the likelihood of false-positive findings and therefore should be interpreted cautiously. Additionally, despite Mayo Clinic being a large tertiary academic center, this is a single-site study, and the population of Rochester is not indicative of large metropolitan areas, which might make study results less generalizable. This restricted our ability to perform a more detailed analysis of race and to explore categories beyond Black, White, and additional racial groups. Gender identity was also not collected, as this was not consistently documented in the EHR. We also had limited data on prehospital interventions, such as restraints or medications applied prior to reaching the ED. As a result of the low rates for both physical and pharmacologic restraints in our data, estimates involving restraints had confidence intervals that tended to be wider. Furthermore, the reasons for prolonged boarding were not always clearly documented in medical records. Therefore, we relied on templated care management notes that offered a more systematic approach to identify these reasons. Nonetheless, this strategy may have not captured all the factors contributing to the prolonged boarding, and it is probable that additional details were undocumented.

## Conclusions

To our knowledge, this is the first study to date exploring ED practices among children and adolescents with a history of OOHP. We found that significant disparities, both in length of stay and physical and pharmacologic restraints use in the ED, exist in this subset of patients. Generally, we believe these findings are robust; however, it is important to note that this disparity was observed after controlling for a few variables, and other confounders may exist that were not included in the model. Future studies should examine these trends in other hospital settings, including medical and surgical units and inpatient psychiatric hospitals.

## Notes

Supplement 1. **eTable 1.** Patient Characteristics (Current OOHP With no OOHP History) **eFigure.** Distribution of Reasons for Prolonged Boarding **eTable 2.** Admissions and Discharges by History of OOHP **eTable 3.** Summaries of Time in ED as Medians With IQRs **eTable 4.** Association of Physical Restraints and Pharmacologic Restraints and Race in Youths With History of OOHP

Supplement 2.
Data Sharing Statement
