# Supplementary material for: Prior Out-of-Home Placement and Length of Stay Among Youths Receiving Mental Health Services in the ED
Source: JAMA Netw Open. 2026 Jan 23;9(1):e2555339. doi: 10.1001/jamanetworkopen.2025.55339 (PMC12831154; doi:10.1001/jamanetworkopen.2025.55339)
Supplement: Supplement 1. — eTable 1. Patient Characteristics (Current OOHP With no OOHP History) eFigure. Distribution of Reasons for Prolonged Boarding eTable 2. Admissions and Discharges by History of OOHP eTable 3. Summaries of Time in ED as Medians With IQRs eTable 4. Association of Physical Restraints and Pharmacologic Restraints and Race in Youths With History of OOHP [file jamanetwopen-e2555339-s001.pdf]

Supplemental Online Content

Kelly CK, Saliba M, Park JH, et al. Prior out-of-home placement and length of stay among youths receiving mental health services. *JAMA Netw Open.* 2026;9(1):e2555339. doi:10.1001/jamanetworkopen.2025.55339

eTable 1. Patient Characteristics (Current OOHP With no OOHP History)

eFigure. Distribution of Reasons for Prolonged Boarding

eTable 2. Admissions and Discharges by History of OOHP

eTable 3. Summaries of Time in ED as Medians With IQRs

eTable 4. Association of Physical Restraints and Pharmacologic Restraints and Race in Youths With History of OOHP

This supplemental material has been provided by the authors to give readers additional information about their work.

© 2026 Kelly CK et al. *JAMA Network Open*.

eTable 1. Patient Characteristics (Current OOHP With no OOHP History)

	Current OOHP (n=25)
Age at ED admit (yrs), median (IQR)	15.8 (14.5, 16.7)
Sex assigned at birth, n (%)	
Female	19 (76)
Male	6 (24)
Race, n (%), N=23	
American Indian/Alaskan Native	1 (4)
Asian	1 (4)
Black or African American	2 (9)
Middle Eastern/North African	0 (0)
Multiracial	0 (0)
White	17 (74)
Other	2 (9)
Ethnicity, n (%), N=24	
Hispanic or Latino	2 (8)
Not Hispanic or Latino	22 (92)
Health insurance, n (%)	
Commercial	11 (44)
Medicaid	14 (56)
Uninsured	0 (0)
Pharmacologic restraints during encounter, n (%)	2 (8)
Intramuscular	2 (100)
Intravenous	0 (0)
Physical restraint during encounter, n (%)	0 (0)
Physical hold	-
Restraint chair	-
Security cuff	-
Soft restraints	-
Presenting concern, n (%)	
Suicidal ideation/self-injurious behavior	15 (60)
Suicide attempt	6 (24)
Violence towards others	2 (8)
Elopement	1 (4)
Substance use	0 (0)
Hallucinations/disorganized	1 (4)
Other	2 (8)
Primary psychiatric diagnosis, n (%)	21 (84)
Bipolar disorder	1 (4)
Psychotic disorder	0 (0)
Autism	0 (0)
Intellectual disability	0 (0)

© 2026 Kelly CK et al. *JAMA Network Open*.

Anxiety disorder	13 (52)
Depressive disorder	18 (72)
ADHD	9 (36)
Other	15 (60)
Previous psychiatric hospitalizations, median (IQR)	0 (0, 2)
Active psychiatric medications, n (%)	
Stimulants	4 (16)
Non-stimulants	3 (12)
Typical antidepressants	12 (48)
Other antidepressants	2 (8)
Neuroleptics	5 (20)
Mood stabilizers	1 (4)
Benzodiazepines	0 (0)
Non-benzodiazepines	3 (12)
Substance use, n (%)	
Cannabis/Marijuana	14 (56)
Nicotine/Tobacco	10 (40)
Alcohol	11 (44)
Cocaine/Amphetamine/stimulant	1 (4)
Anxiolytic/hypnotics/sedatives	2 (8)
Opioid	0 (0)
Other	1 (4)
History of trauma, n (%)	19 (76)
Family conflicts	7 (28)
Physical abuse	7 (28)
Neglect	4 (16)
Medical trauma	0 (0)
Sexual trauma	8 (32)
Homelessness	0 (0)
Emotional abuse	3 (12)
Verbal abuse	6 (24)
War trauma	0 (0)
Natural disaster	0 (0)
Other	0 (0)
Legal history, n (%)	6 (24)
Sexually intrusive behavior, n (%)	0 (0)
ED discharge location, n (%)	
Medical hospital	1 (4)
Inpatient psychiatric hospital	20 (80)
Residential treatment center	0 (0)
Juvenile detention center	2 (8)
Foster care	0 (0)

© 2026 Kelly CK et al. *JAMA Network Open*.

Home with family	1 (4)
c	1 (4)
Legal guardian, n (%)	
Mother	18 (72)
Father	8 (32)
Stepparent	0 (0)
Other family member	3 (12)
Adoptive parent	0 (0)
Case manager/county	1 (4)
Other	1 (4)

‘Other’ race is an EHR category that patients self-report

eFigure. Distribution of Reasons for Prolonged Boarding

	Overall n=1597 (%)
Acuity aggression	170 (11)
Assessment length	170 (11)
Awaiting safe disposition	268 (17)
Bed availability	424 (27)
Medical clearance	231 (14)
Unable to reach guardian	31 (2)
Not prolonged	475 (30)

158 patients had more than one reason for prolonged boarding

14 patients had three reasons: 11 had acuity/aggression & bed availability & medical clearance, 2 had acuity/aggression & bed availability & unable to reach guardian, and 1 had awaiting safe disposition & medical clearance & unable to reach guardian

© 2026 Kelly CK et al. *JAMA Network Open*.

eTable 2. Admissions and Discharges by History of OOHP

	Psychiatric Admission	Medical Admission	Discharged
History of OOHP	140	11	177
No history of OOHP	864	16	364

© 2026 Kelly CK et al. *JAMA Network Open*.

eTable 3. Summaries of Time in ED as Medians With IQRs

	Time in ED (hrs)
Out of home placement	15.9 (5.1, 29.6)
No out of home placement	4.8 (3.6, 10.7)
Race	
Black or African American	16.0 (4.4, 33.2)
Other	18.3 (5.9, 27.6)
White	15.0 (5.0, 29.8)
Ethnicity	
Hispanic or Latino	21.6 (10.6, 43.1)
Not Hispanic or Latino	14.9 (5.0, 28.4)

‘Other’ includes: American Indian/Alaskan Native, Asian, Middle Eastern/North African, Multiracial and Other race subcategories

© 2026 Kelly CK et al. *JAMA Network Open*.

eTable 4. Association of Physical Restraints and Pharmacologic Restraints and Race in Youths With History of OOHP

	Unadjusted			Adjusted		
	OR	95% CI	p-value	OR	95% CI	p-value
Physical restraints						
Race						
Black or African American	2.44	(0.87, 6.86)	0.09	3.51	(0.79, 15.59)	0.10
Other	0.45	(0.06, 3.55)	0.45	0.49	(0.04, 5.33)	0.55
Ethnicity						
Hispanic or Latino	3.16	(1.13, 8.81)	0.028	2.27	(0.49, 10.51)	0.29
Pharmacologic restraints						
Race						
Black or African American	3.30	(1.40, 7.81)	0.007	4.02	(1.25, 12.95)	0.02
Other	0.33	(0.04, 2.56)	0.29	0.27	(0.03, 2.67)	0.26
Ethnicity						
Hispanic or Latino	1.89	(0.72, 5.00)	0.20	1.52	(0.39, 5.84)	0.54

Reference group for out of home placement is no. Reference group for race is White. Reference group for ethnicity is not Hispanic or Latino.

'Other' includes: American Indian/Alaskan Native, Asian, Middle Eastern/North African, Multiracial and Other race subcategories

Adjusted for age at admission, sex, insurance, number of prior diagnoses, number of psychotropic medications, number of psychiatric hospitalizations, history of trauma, presenting concern(s), and reason(s) for prolonged boarding.

© 2026 Kelly CK et al. *JAMA Network Open*.
