# Supplementary material for: Prior Out-of-Home Placement and Length of Stay Among Youths Receiving Mental Health Services in the ED
Source: JAMA Netw Open. 2026 Jan 23;9(1):e2555339. doi: 10.1001/jamanetworkopen.2025.55339 (PMC12831154; doi:10.1001/jamanetworkopen.2025.55339)
Supplement: Supplement 2. — Data Sharing Statement [file jamanetwopen-e2555339-s002.pdf]

Data Sharing Statement

Kelly. Prior Out-of-Home Placement and Length of Stay Among Youths Receiving Mental Health Services. *JAMA Netw Open*. Published January 23, 2026. doi:10.1001/jamanetworkopen.2025.55339

Data
Data available: No
